# Contextualised high-intensity running profiles of elite football players with reference to general and specialised tactical roles

**DOI:** 10.5114/biolsport.2023.116003

**Published:** 2022-05-10

**Authors:** Wonwoo Ju, Dominic Doran, Richard Hawkins, Mark Evans, Andy Laws, Paul S Bradley

**Affiliations:** 1 The Research Institution for Sport and Exercise Sciences at Liverpool John Moores University, Liverpool, England, UK; 2 Football Medicine and Science Department at Manchester United Football Club, Manchester, UK; 3 Department of Computer Science, Liverpool John Moores University, Liverpool, UK; 4 Football Science Consultant, UK

**Keywords:** Match performance, Physical-tactical data, High-intensity, Positions, Soccer

## Abstract

The present study aimed to contextualise physical metrics with tactical actions according to general and specialised tactical roles. A total of 244 English Premier League players were analysed by coding player’s physical-tactical actions via the fusion of tracking data and video. Data were analysed across 5 general (Central Defensive Players = CDP, Wide Defensive Players = WDP, Central Midfield Players = CMP, Wide Offensive Players = WOP, Central Offensive Players = COP) and 11 specialised positions (Centre Backs = CB, Full-Backs = FB, Wing-Backs = WB, Box-to-Box Midfielders = B2BM, Central Defensive Midfielders = CDM, Central Attacking Midfielders = CAM, Wide Midfielders = WM, Wide Forwards = WF, Centre Forwards = CF). COP covered more distance at high-intensity (> 19.8 km · h^-1^) when performing actions such as ‘Break into Box’, Run in Behind/Penetrate’, and ‘Close Down/Press’ than other positions (ES: 0.6–5.2, P < 0.01). WOP covered more high-intensity ‘Run with Ball’ distance (ES: 0.7–1.7, P < 0.01) whereas WDP performed more ‘Over/Underlap’ distance than other positions (ES: 0.9–1.4, P < 0.01). CDP and WDP covered more high-intensity ‘Covering’ distances than other positions (ES: 0.4–2.4, P < 0.01). Nonetheless, data demonstrated that implementing specialised positional analysis relative to a generalised approach is more sensitive in measuring physical-tactical performances of players with the latter over or underestimating the match demands of the players compared to the former. A contextualised analysis may assist coaches and practitioners when designing position or even player-specific training drills since the data provides unique physical-tactical trends across specialised roles.

## INTRODUCTION

As technology plays a more prominent role in modern football (soccer), the reliance on tracking-based technologies has increased exponentially [1]. Due to the complex nature of football, researchers have typically adopted a reductionist approach analysing either physical or technical metrics in isolation [2]. Despite this, a great deal of research has quantified the physical demands of elite players during matches and examined how this is affected by other factors such as positions, formations, and opponent standard [3–5]. Longitudinal match performance data trends emphasise that the distances covered at high-intensity have increased by ˜20–30% with only a 2–4% increase in the total distance covered over the last decade [6, 7]. Consequently, greater attention has been paid to high-intensity actions as it helps practitioners to prepare players for the physical demands of modern match-play through benchmarking contemporary match-play requirements during training sessions [8].

High-intensity running profiles of various playing positions have been well documented [3, 9, 10] and used by coaches and practitioners to target modern football requirements that can be tailored according to different roles [11]. Nevertheless, the vast majority of studies in the scientific literature defined positional roles generically such as defenders, midfielders, and attackers [12, 13] or in terms of the general positions such as centre backs, full-backs, central midfielders, wide midfielders, and forwards [3, 10]. This generalised positional analysis limits our understanding of the true physical demands of players with more specialised tactical roles (e.g., central defensive or attacking midfielders) during a match [14]. Additionally, based on previous findings [10, 14, 15], one could assume that using a generalised positional analysis may be less sensitive in detecting the true physical-tactical match demands compared to a specialised positional method. Thus, research that compares general versus specialised positions is warranted to identify whether disparities exist between the two different positional analyses.

Limited research has quantified the physical demands of elite players using a specialised playing position analysis. It has been reported that central attacking midfielders covered ˜10–30% more high-intensity distance than central defensive midfielders [15, 16]. However, the methods of differentiating central midfield players into specialised roles in these studies were not disclosed, thus confounding study replication. Konefal and colleagues attempted to quantify performance profiles of specialised tactical roles using heat maps [17]. Yet, this study failed to differentiate full-backs (FB) and wing-backs (WB). Others have determined the tactical roles of wide defensive players (WDP) based on positions that are predefined within a playing system or formation [18, 19]. For instance, FB are based on a formation with four players at the back (e.g., 4-3-3 formations) and WB three at the back (e.g., 3-4-3 formations). The definitions of various wide defender subsets are not objectively defined but could relate to FB performing a more defensive role whilst WB could be regarded as a mixture of a FB and a winger due to dual responsibilities [8, 20]. Hence, differentiating player positions with specialised tactical roles should be accomplished by the observation of each player for the duration of match-play in addition to other analytical modalities (heat maps, average position etc.) to detect the true tactical role/playing style of players during match-play.

Although physical metrics provide some insight to practitioners, it is questionable how receptive coaches are to this basic data [2]. As coaches can sometimes have difficulty communicating with practitioners [11], especially in relation to data that are not contextualised appropriately. This seems to be due to researchers typically asking ‘WHAT’ distance players covered [2]. Therefore, analysing ‘WHY’ players cover that distance is very much warranted since the ‘WHY’ explains the modulators of the physical efforts. Recently, a systematic methodology that amalgamates physical metrics alongside their tactical purposes has been developed [21]. This approach may help coaches and practitioners understand the physical data contextualised to the tactical dynamics, thus allowing more practical application. Combining this approach with detailed specialised tactical roles would provide additional insights of individual’s physical-tactical demands. Thus, the present study aimed to contextualise physical data with the key tactical purposes of the actions undertaken according to both their general and specialised tactical roles in which comparisons were made between them to determine their sensitivity in measuring physical-tactical performance during match-play.

## MATERIALS AND METHODS

### Match Analysis and Player Data

Match physical-tactical data were derived from the 2018–19 English Premier League season using an integrated approach and a novel filter developed for this research. Players’ actions were captured by cameras placed at roof level during matches, and their physical-tactical actions were manually coded by using the integrated approach. The validity and reliability of this approach have been previously verified and additional information regarding the data provider and filter used can also be found in this source [21]. The validity of the novel method demonstrated a strong agreement between the responses of both UEFA licensed coaches and performance analysts versus the gold standard responses (˜92%), and its inter- and intra-observer reliability was a strong (κ = 0.81) to almost perfect (κ = 0.94), respectively. The new filter isolated high-intensity activities reaching speeds > 19.8 km · h^-1^ for a minimal dwell time of 1 s [22].

The researcher completed 350 hours of coding to analyse 50 competitive matches. This consisted of the total number of 388 individual outfield players across 1,265 player observations. However, only outfield players who had completed the entire match in the same position were included (244 players across 583 player observations for the analyses of general positions). This consisted of Central Defensive Players (CDP, *n* = 179), Wide Defensive Players (WDP, *n* = 147), Central Midfield Players (CMP, *n* = 167), Wide Offensive Players (WOP, *n* = 54), and Central Offensive Players (COP, *n* = 36). However, for the analysis of specialised positions, 8 players were excluded since it was ambiguous to sub-categorise them into a specialised tactical role, thus this included 236 players across 529 player observations. This resulted in Centre Backs (CB^2^, two at the back, *n* = 130; CB^3^, three at the back, *n* = 49), Full-Backs (FB, *n* = 39), Wing-Backs (WB, *n* = 70), Box-to-Box Midfielders (B2BM, *n* = 94), Central Defensive Midfielders (CDM, *n* = 49), Central Attacking Mid-fielders (CAM, *n* = 11), Wide Midfielders (WM, *n* = 40), Wide Forwards (WF, *n* = 14), and Centre Forwards (CF^1^, one centre forward, *n* = 14; or CF^2^, two centre forwards, *n* = 22). All data were analysed for the duration of each half, including stoppage time. Prior to analysis, all original data were anonymised to ensure confidentiality. Ethical approval was granted by Liverpool John Moores University (19/SPS/027) research ethics committee.

### Match Control and Data Balance

Matches were randomly selected whilst simultaneously controlling various situational factors (phases of season, locations, and team or opponent standards) in line with previously outlined approaches [6]. Therefore, the number of matches for each factor was initially balanced. Matches were included only if they were close games (goal differential ≤ 2) and were excluded if there was a player dismissal as this can impact overall work-rates [23].

### Demarcation of Player’s Tactical Roles

**FIG. 1:**
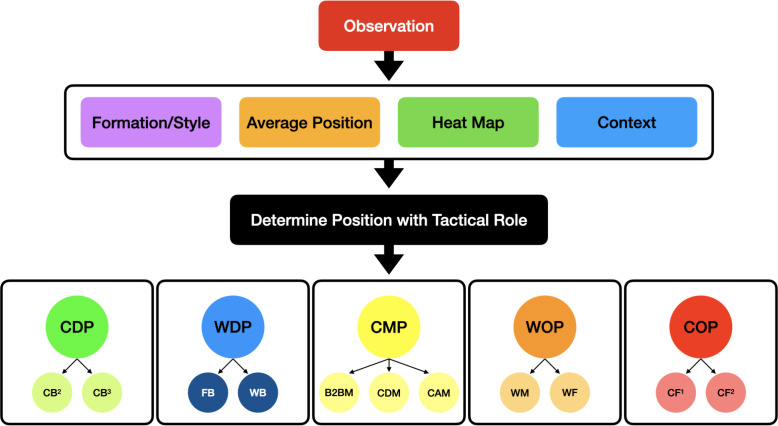
The systematic process of determining a player’s tactical role in the team. CDP: Central Defensive Players (CB2: two Centre Backs, CB3: three Centre Backs), WDP: Wide Defensive Players (FB: Full-backs, WB: Wing-backs), CMP: Central Midfield Players (B2BM: Box-to-Box Midfielders, CDM: Central Defensive Midfielders, CAM: Central Attacking Midfielders), WOP: Wide Offensive Players (WM: Wide Midfielders, WF: Wide Forwards), COP: Central Offensive Players (CF1: one Centre Forward, CF2: two Centre Forwards).

**FIG. 2:**
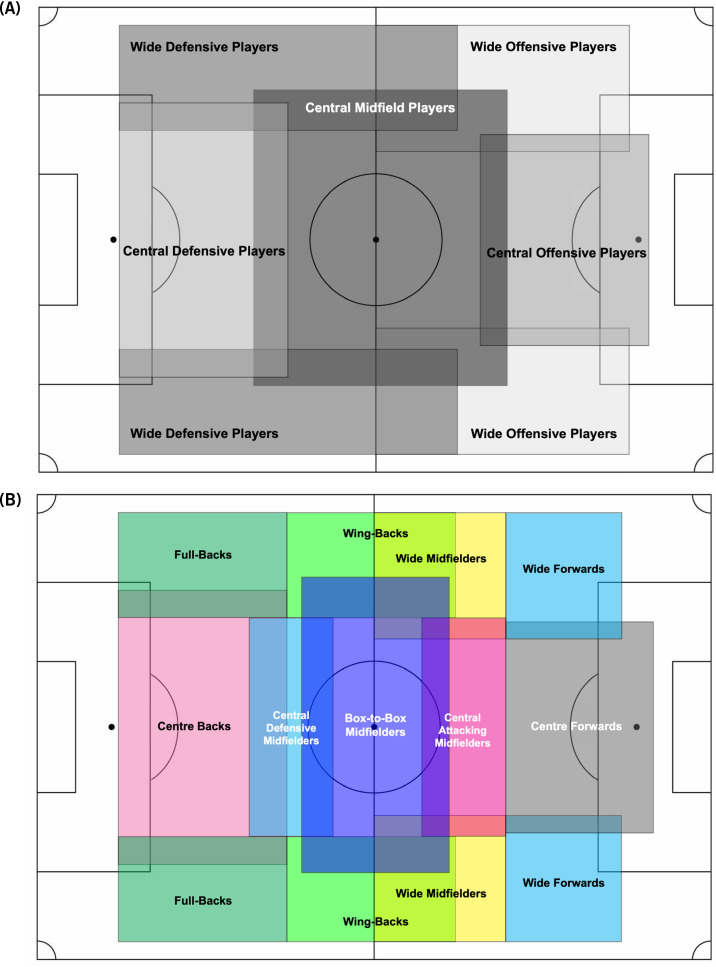
General (A) and specialised (B) tactical roles based on match analyses. Adapted from Aalbers and Van Haaren [24].

A systematic approach was applied to the demarcation between various tactical roles using descriptors of general and specialised roles (Figure 1). The methodology of differentiating specialised positions was adapted from previous research [24]. Once outfield players were assigned to one of the five general positions (Figure 2A), they were then specifically sub-categorised according to their specific playing style/formation (Figure 2B). As various situational factors have an influence on the style of play that can be modulated by different tactical roles [25], context was considered whilst using a player’s average position and heat map in an attempt to determine player’s relevant tactical role in the team. This was verified by observing video footage of the entire match.

Inter-rater reliability for differentiating specialised tactical roles was assessed by two observers (UEFA qualified coach and the researcher) watching the entire match of players for each specialised tactical role (*n* = 55). The kappa statistic of 0.84 reflects a strong level of inter-observer consistency. Intra-observer reliability test undertaken by the researcher resulted in the kappa statistic value of 1.00, which is interpreted as a perfect intra-observer reliability [26].

### The Integrated Approach of Match Performance

High-intensity actions isolated by the new filter were synchronised with video footage of each player throughout matches to code the tactical purpose of each action. All coding occurred using QuickTime Player (Apple Inc, Cupertino, California) to watch video and then categorise tactical actions (Table 1).

**TABLE 1:** Descriptions of the variables utilised within the integrated approach.

Variables	Description
***In Possession***	

Run with Ball	Player moves with the ball either dribbling with small touches or running at speed with the fewer ball touches.
Over/Underlap	Player runs from behind to in front of the player on the ball or receiving the ball.
Push up Pitch	Player moves up the pitch to play offside and/or to squeeze to a higher line.
Break into Box	Player enters the opposition’s penalty box to receive the ball (typically receive ball from a cross – ball in front and wide).
Run in Behind/Penetrate	Player attacks space behind, overtakes and/or unbalances the opposition defence (typically ball is behind).
Move to Receive/Exploit Space	Player moves to receive a pass from a teammate or to create/exploit space (typically come short or move wide to receive ball).
Support Play	Player supports from behind/level by trying to engage in offensive/transition play (typically during fast transitions).

***Out of Possession***	

Close Down/Press	Player runs directly towards opposition player on or receiving the ball, or towards space or players not on/receiving the ball.
Interception	Player cuts out pass.
Recovery Run	Player runs back towards their own goal to be goal side when out of position.
Covering	Player moves to cover space or an opposition player while remaining goal side.

***Unclassifiable***	

Other	All other variables that could not be categorised by the above.

The coding process was as follows: high-intensity actions with one tactical action were classified as a single action with dual tactical actions being classified as a hybrid action. High-intensity actions with more than three tactical actions were coded as ‘Other’. If the high-intensity action consists of 70–90% of the primary and 10–30% of the secondary action, it was classified as a hybrid action. However, if it is made up of 50–60% of the primary and 40–50% of the secondary action, then it was classified as ‘Other’. As hybrid actions are a combination of the primary and the secondary actions [2], single action events and the primary tactical movements of the hybrid actions were merged to simplify data outputs.

### Statistical Analyses

Data are presented as the mean ± standard deviation. All statistical analyses were conducted using IBM SPSS Statistics for Mac OS X, version 26 (IBM Corp., Armonk, N.Y., USA). Data normality was verified by Shapiro-Wilk and Kolmogorov-Smirnov tests. One-way analyses of variance (ANOVA) were used to compare each position with Bonferroni post hoc test used to determine localised differences. Statistical significance was set at *P* < 0.05. Effect size (ES) for the meaningfulness of the difference was determined as follows: trivial (≤ 0.2), small (> 0.2–0.6), moderate (> 0.6–1.2), large (> 1.2–2.0) and very large (> 2.0–4.0) [27]. The coefficient of variation (CV) was analysed for match-to-match variabilities of general and specialised positions [28].

## RESULTS

### General Tactical Roles

In possession COP covered 62–1,434% and 88–32,767% more high-intensity distances for ‘Break into Box’ and ‘Run in Behind/ Penetrate’ than other positions (ES: 0.6–2.8 and 1.1–5.2, respectively, *P* < 0.01) whilst WOP covered 71–323% more ‘Run with Ball’ distance than other positions (ES: 0.7–1.7, *P* < 0.01). WDP and WOP covered 35–8,254% and 38–748% greater high-intensity distances for ‘Support Play’ and ‘Move to Receive/Exploit Space’, respectively, than CDP and CMP (ES: 0.3–2.9, *P* < 0.05) with the former covering 548–5,340% more ‘Over/Underlap’ distance than other positions (ES: 0.9–1.4, *P* < 0.01).

**FIG. 3:**
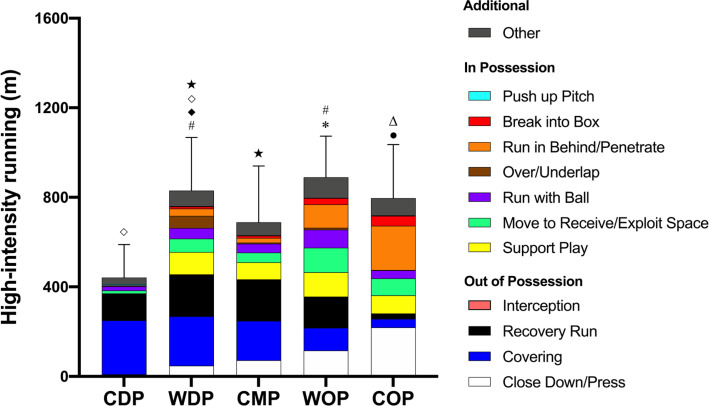
Contextualised distances at high-intensity running covered by general positions. ^●^More distance for ‘Break into Box’ and ‘Run in Behind/Penetrate’ than others (*P*<0.01). ^*^More distance for ‘Run with Ball’ than others (*P*<0.01). ^#^More distance for ‘Support Play’ and ‘Move to Receive/Exploit Space’ than CDP and CMP (*P*<0.05). ^◆^ More distance for ‘Over/Underlap’ than others (P<0.01). ^Δ^More distance for Close Down/Press’ than others (*P*<0.01). ^◇^More distance for ‘Covering’ than CMP, WOP, and COP (*P*<0.01). ^★^More distance for ‘Recovery Run’ than CDP, WOP, and COP (*P*<0.01). ‘Interception’ and ‘Push up Pitch’ were very infrequent, thus not visualised on figure.

Out of possession COP ran 89–2,307% greater high-intensity ‘Close Down/Press’ distance than other positions (ES: 1.1–4.2, *P* < 0.01). CDP and WDP performed 25–532% more high-intensity ‘Covering’ distance than other positions (ES: 0.4–2.4, *P* < 0.01) whilst WDP and CMP covered 34–670% more ‘Recovery Run’ distance than other positions (ES: 0.5–1.8, *P* < 0.05). Contextualised high-intensity distance for general positions are presented in Figure 3.

### Specialised Tactical Roles

In possession, WB, B2BM, CAM, and WM covered 535–51,567% greater high-intensity ‘Support Play’ distance than CB^2^, CB^3^ and CDM (ES: 1.0–5.0, *P* < 0.01). WB performed 103–16,925% more high-intensity ‘Over/Underlap’ distances than other positions (ES: 0.6–1.8, *P* < 0.01).

**FIG. 4:**
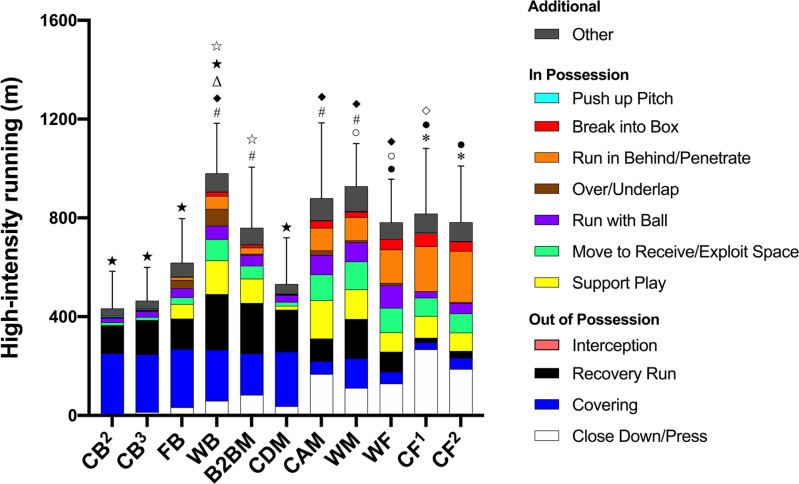
Contextualised distances at high-intensity running covered by specialised positions. *More distance for ‘Run in Behind/Penetrate’ than CB^2^, CB^3^, FB, WB, B2BM, CDM, and WM (*P*<0.01). ^●^More distance for ‘Break into Box’ than CB^2^, CB^3^, FB, WB, B2BM, CDM, and WM (*P*<0.05). ^○^More distance for ‘Run with Ball’ than CB^2^, CB^3^, FB, B2BM, CDM, CF^1^ (*P*<0.01). ^#^More distance for ‘Support Play’ than CB^2^, CB^3^, and CDM (*P*<0.01). ^◆^More distance for ‘Move to Exploit Space/Receive’ than CB^2^, CB^3^, FB, and CDM (*P*<0.01). ^Δ^More distance for ‘Over/Underlap’ than others (*P*<0.01). ^◇^More distance for ‘Close Down/Press’ than others (*P*<0.01). ^★^More distance for ‘Covering’ than CAM, WM, WF, CF^1^, and CF^2^ (*P*<0.01). ^☆^More distance for ‘Recovery Run’ than CB^2^, CB^3^, FB, CAM, WF, CF^1^, and CF^2^ (*P*<0.01). ‘Interception’ and ‘Push up Pitch’ were very infrequent, thus not visualised on figure.

Out of possession, CF^1^ performed 43–3,621% greater high-intensity ‘Close Down/Press’ distances than other positions (ES: 0.7–5.4, *P* < 0.01) while defensive players (CB^2^, CB^3^, FB, WB, and CDM) covered 73–796% greater ‘Covering’ distance than offensive players (CAM, WM, WF, CF^1^, and CF^2^; ES: 1.0–2.6, *P* < 0.01). Figure 4 illustrates contextualised high-intensity distance for specialised positions.

### Comparison between Specialised Tactical Roles and their General Position

FB covered 34% less high-intensity distance (618 ± 178 m, ES: 0.9, *P* < 0.01) whilst WB covered 15% more distance (981 ± 203 m, ES: 0.7, *P* < 0.01) when compared to WDP (830 ± 238 m). CDM covered 30% less distance in high-intensity running (532 ± 187 m, ES: 0.7, *P* < 0.01) whilst CAM performed 22% more distance than CMP albeit no statistical difference (880 ± 305 m vs 689 ± 251 m, respectively, *P* > 0.05). Table 2 depicts the average distance and duration per physical-tactical action with the average number of activities per match performed by general and specialised positions.

**TABLE 2:** Average distance and duration per action with average number of actions per match across various tactical roles.

Type	Position	Variable	In Possession						Out of Possession			Overall
			SP	MTR/ES	OVL/UDL	RWB	RIB/PEN	BIB	CD/PRE	COV	RR	
General	CDP (*n* = 163)	Distance (m)	26 ± 19	18 ± 8	36 ± 11	17 ± 8	18 ± 6	14 ± 7	15 ± 5	18 ± 8	26 ± 15	20 ± 11
		Duration (sec)	3 ± 3	2 ± 1	4 ± 2	2 ± 1	2 ± 1	1 ± 1	1 ± 1	2 ± 1	3 ± 2	2 ± 1
		Actions (No.)	0 ± 0	1 ± 1	0 ± 0	1 ± 2	0 ± 0	0 ± 0	1 ± 1	13 ± 4	4 ± 3	23 ± 6

Specialised	CB^2^ (*n* = 130)	Distance (m)	28 ± 19	18 ± 8	26 ± 8	17 ± 7	16 ± 6	16 ± 9	15 ± 5	18 ± 8	27 ± 15	20 ± 11
		Duration (sec)	3 ± 3	2 ± 1	3 ± 1	2 ± 1	2 ± 1	2 ± 1	1 ± 1	2 ± 1	3 ± 2	2 ± 1
		Actions (No.)	0 ± 0	1 ± 1	0 ± 0	1 ± 2	0 ± 0	0 ± 0	0 ± 1	14 ± 4	4 ± 2	22 ± 7

	CB^3^ (*n* = 49)	Distance (m)	13	17 ± 9	43 ± 6	19 ± 9	21 ± 6	12 ± 2	15 ± 5	19 ± 9	26 ± 14	20 ± 11
		Duration (sec)	1	2 ± 1	5 ± 1	2 ± 1	3 ± 1	1 ± 0	1 ± 1	2 ± 1	3 ± 2	2 ± 1
		Actions (No.)	0 ± 0	1 ± 1	0 ± 0	1 ± 2	0 ± 0	0 ± 1	1 ± 1	12 ± 4	5 ± 3	23 ± 6

General	WDP (*n* = 120)	Distance (m)	24 ± 13	20 ± 10	28 ± 14	22 ± 12	20 ± 9	19 ± 7	15 ± 5	20 ± 10	27 ± 14	22 ± 12
		Duration (sec)	3 ± 2	2 ± 1	3 ± 2	2 ± 2	2 ± 1	2 ± 1	1 ± 1	2 ± 1	3 ± 2	2 ± 2
		Actions (No.)	4 ± 3	3 ± 3	2 ± 2	2 ± 2	2 ± 2	0 ± 1	3 ± 2	11 ± 4	7 ±4	38 ± 10

Specialised	FB (*n* = 39)	Distance (m)	22 ± 13	19 ± 8	28 ± 13	21 ± 12	21 ± 11	0	15 ± 5	20 ± 10	24 ± 12	21 ± 11
		Duration (sec)	2 ± 2	2 ± 1	3 ± 2	2 ± 2	2 ± 1	0	1 ± 1	2 ± 1	3 ± 2	2 ± 2
		Actions (No.)	3 ± 2*	1 ± 1*	1 ± 1	2 ± 1	1 ± 1*	0*	2 ± 2	12 ± 4	5 ± 3*	30 ± 7*

	WB (*n* = 70)	Distance (m)	25 ± 14	20 ± 11	28 ± 15	22 ± 12	20 ± 8	18 ± 7	15 ± 5	20 ± 10	27 ± 14	22 ± 12
		Duration (sec)	3 ± 2	2 ± 1	3 ± 2	2 ± 2	2 ± 1	2 ± 1	1 ± 1	2 ± 1	3 ± 2	2 ± 2
		Actions (No.)	5 ± 3****	4 ± 3****	2 ± 2	3 ± 2	3 ± 2****	1 ± 1***	4 ± 3	11 ± 4	8 ±4	45 ± 8****

General	CMP (*n* = 132)	Distance (m)	23 ± 13	19 ± 9	22 ± 16	20 ± 10	20 ± 10	19 ± 10	16 ± 7	20 ± 10	24 ± 13	21 ± 11
		Duration (sec)	3 ± 2	2 ± 1	2 ± 2	2 ± 1	2 ± 1	2 ± 1	2 ± 1	2 ± 1	3 ± 2	2 ± 2
		Actions (No.)	3 ±4	2 ± 2	0 ± 1	2 ± 2	1 ± 2	1 ± 1	4 ± 4	9 ± 5	8 ±4	34 ± 11

Specialised	B2BM *(n* = 94)	Distance (m)	23 ± 14	19 ± 10	21 ± 10	21 ± 10	21 ± 10	19 ± 10	16 ± 7	20 ± 10	25 ± 14	21 ± 12
		Duration (sec)	3 ± 2	2 ± 1	2 ± 1	2 ± 1	2 ± 1	2 ± 1	2 ± 1	2 ± 1	3 ± 2	2 ± 2
		Actions (No.)	4 ±4	3 ± 2	0 ± 1	2 ± 2	1 ± 1	1 ± 1	5 ± 3	9 ± 5	8 ±4	37 ± 11

	CDM (*n* = 46)	Distance (m)	23 ± 13	16 ± 6	13 ± 3	19 ± 10	22 ± 4	28 ± 22	15 ± 6	20 ± 10	23 ± 12	20 ± 11
		Duration (sec)	3 ± 2	2 ± 1	1 ± 0	2 ± 1	3 ± 1	3 ± 3	1 ± 1	2 ± 1	3 ± 2	2 ± 2
		Actions (No.)	1 ± 1*	1 ± 1*	0 ± 0	1 ± 1	0 ± 0*	0 ± 0*	2 ± 0*	11 ± 5	7 ± 3	26 ± 8*

	CAM (*n* = 11)	Distance (m)	23 ± 11	19 ± 9	30 ± 27	23 ± 11	19 ± 9	17 ± 7	18 ± 8	20 ± 12	22 ± 13	20 ± 11
		Duration (sec)	3 ± 2	2 ± 1	3 ± 3	3 ± 2	2 ± 1	2 ± 1	2 ± 1	2 ± 2	3 ± 2	2 ± 1
		Actions (No.)	7 ± 4****	6 ± 3****	1 ± 1	3 ± 3	5 ± 4****	2 ± 1****	9 ± 8****	3 ± 2*	4 ± 2*	43 ± 14***

General	WOP (*n* = 40)	Distance (m)	23 ± 12	22 ± 12	21 ± 8	22 ± 12	21 ± 10	20 ± 8	18 ± 8	21 ± 10	24 ± 13	21 ± 11
		Duration (sec)	3 ± 2	2 ± 2	2 ± 1	2 ± 2	2 ± 1	2 ± 1	2 ± 1	2 ± 1	3 ± 2	2 ± 2
		Actions (No.)	5 ± 3	5 ± 3	0 ± 1	4 ± 2	5 ± 4	1 ± 1	7 ± 3	5 ± 3	6 ± 3	42 ± 7

Specialised	WM (*n* = 40)	Distance (m)	23 ± 12	22 ± 12	22 ± 8	23 ± 12	22 ± 11	19 ± 8	17 ± 8	21 ± 10	24 ± 13	22 ± 11
		Duration (sec)	3 ± 2	2 ± 2	2 ± 1	3 ± 2	2 ± 1	2 ± 1	2 ± 1	2 ± 1	3 ± 2	2 ± 2
		Actions (No.)	5 ± 3	5 ± 3	0 ± 1	3 ± 2	4 ± 4	1 ± 1	6 ± 3	6 ± 3	6 ± 3	44 ± 7

	WF (*n* = 14)	Distance (m)	22 ± 11	20 ± 10	18 ± 6	20 ± 10	19 ± 9	20 ± 9	19 ± 9	20 ± 9	24 ± 13	20 ± 10
		Duration (sec)	2 ± 2	2 ± 1	2 ± 1	2 ± 1	2 ± 1	2 ± 1	2 ± 1	2 ± 1	3 ± 2	2 ± 1
		Actions (No.)	4 ± 2	5 ± 3	1 ± 1	5 ± 2	7 ± 4	2 ± 1	7 ± 4	2 ± 2*	3 ± 3	39 ± 7

General	COP (*n* = 23)	Distance (m)	29 ± 16	22 ± 11	24 ± 5	22 ± 11	21 ± 10	19 ± 7	20 ± 10	20 ± 9	25 ± 14	21 ± 11
		Duration (sec)	3 ± 2	2 ± 2	3 ± 1	2 ± 2	2 ± 1	2 ± 1	2 ± 1	2 ± 1	3 ± 2	2 ± 2
		Actions (No.)	3 ± 2	4 ± 3	0 ± 0	2 ± 2	9 ± 4	2 ± 2	11 ± 6	2 ± 2	1 ± 1	38 ± 10

Specialised	CF^1^(*n* = 14)	Distance (m)	29 ± 16	22 ± 11	0	25 ± 15	21 ± 9	19 ± 8	20 ± 11	19 ± 8	32 ± 11	21 ± 11
		Duration (sec)	3 ± 2	3 ± 2	0	3 ± 2	2 ± 1	2 ± 1	2 ± 2	2 ± 1	4 ± 2	2 ± 2
		Actions (No.)	3 ± 2	3 ± 2	0	1 ± 1	9 ± 3	3 ± 2	13 ± 7	1 ± 1	1 ± 1	38 ± 11

	CF^2^(*n* = 22)	Distance (m)	29 ± 16	22 ± 12	24 ± 5	21 ± 10	22 ± 11	18 ± 7	19 ± 10	20 ± 9	23 ± 14	21 ± 11
		Duration (sec)	3 ± 2	2 ± 2	3 ± 1	2 ± 1	2 ± 1	2 ± 1	2 ± 1	2 ± 1	3 ± 2	2 ± 2
		Actions (No.)	3 ± 2	4 ± 3	0 ± 0	2 ± 2	10 ± 5	2 ± 1	10 ± 4	2 ± 3	1 ± 1	38 ± 9

General	Overall	Distance (m)	24 ± 14	20 ± 10	27 ± 14	21 ± 11	21 ± 10	19 ± 8	17 ± 8	19 ± 9	25 ± 14	21 ± 11
		Duration (sec)	3 ± 2	2 ± 1	3 ± 2	2 ± 2	2 ± 1	2 ± 1	2 ± 1	2 ± 1	3 ± 2	2 ± 2
		Actions (No.)	3 ± 3	2 ± 3	1 ± 1	2 ± 2	2 ± 3	1 ± 1	4 ± 4	10 ± 5	6 ± 4	33 ± 12

Specialised	Overall	Distance (m)	24 ± 14	20 ± 10	27 ± 14	21 ± 11	21 ± 10	18 ± 8	17 ± 8	19 ± 9	25 ± 15	21 ± 11
		Duration (sec)	3 ± 2	2 ± 1	3 ± 2	2 ± 2	2 ± 1	2 ± 1	2 ± 1	2 ± 1	3 ± 2	2 ± 2
		Actions (No.)	3 ± 3	2 ± 3	1 ± 1	2 ± 2	2 ± 3	1 ± 1	4 ± 4	10 ± 5	6 ± 4	32 ± 12

General Positions: CDP, Central Defensive player; WDP, Wide Defensive player; CMP, Central Midfield player; WOP, Wide Offensive player; COP, Central Offensive player. Specialised Positions: CB^2^, two Centre Backs; CB^3^, three Centre Backs; FB, Full-backs; WB, Wing-backs; B2BM, Box-to-Box Midfielders; CDM, Central Defensive Midfielders; CAM, Central Attacking Midfielders; WM, Wide Midfielders; WF, Wide Forwards; CF^1^, one Centre Forward; CF^2^, two Centre Forwards. SP: ‘Support Play’, MTR/ES: ‘Move to Receive/ Exploit Space’, OVL/UDL: ‘Overlap/Underlap’, RWB: ‘Run with Ball’, RIB/PEN: ‘Run in Behind/Penetrate’, BIB: ‘Break into Box’, CD/ PRE: ‘Close Down/Press’, COV: ‘Covering’, RR: ‘Recovery Run’. ‘Push up Pitch’ and ‘Interception’ were excluded as no differences were found between all positions. Values are means and standard deviations.

* Less number of actions per match than their general position (*P* < 0.01).

** Less number of actions per match than their general position (*P* < 0.05).

*** Greater number of actions per match than their general position (*P* < 0.05).

**** Greater number of actions per match than their general position (*P* < 0.01).

### Match-to-Match Variability

The mean percentages of CVs for high-intensity distances produced by general and specialised positions were 22 ± 13% and 21 ± 14%, respectively, whilst those for the contextualised actions were 67 ± 25% and 62 ± 29%, respectively, regardless of physical-tactical variables.

## DISCUSSION

The present study is the first to contextualise physical performance profiles of elite players with tactical activities executed across various tactical roles, whereby comparisons were made between general and specialised positions to determine disparities between them. Players’ physical-tactical demands of play are significantly under or overestimated if adopting generalist positions (e.g., CMP, WDP and etc.), thus using a specialised positional analysis is critical to improving the sensitivity of player match performance. Data provides insights into ‘WHY’ players cover the high-intensity running distance during matches, which can ultimately help coaches and practitioners to design position- or even player-specific training drills. However, the reader should be aware that the match-to-match variability for high-intensity running distance (CV: 21–22%) and physical-tactical actions (CV: 62–67%) were high, which agrees with previous findings [29–32]. This could indicate that these context-based parameters are sensitive to the way teams set up tactically from game to game but also how each team modulates their own running performance and that of the opposition.

Previous studies demonstrated that the physical demand of match-play was highly dependent on playing positions with central defenders covering the lowest high-intensity running distance and wide mid-fielders the greatest [3, 10], which is in accordance with the findings of the present study. Interestingly, the studies above revealed that high-intensity running distance covered by wide midfielders (˜1000–1200 m) was greater than that covered by wide defenders (˜900–1000 m) although the present study demonstrated that there was no difference between WDP and WOP (˜830 m vs ˜890 m). Some discrepancies in the distance covered may occur between studies possibly due to different filtering methods and dwell times adopted [33]. That being said, it is more likely because the playing style of WDP has evolved from the traditional FB to WB in modern football, especially in the English Premier League where the physical demands of match-play have increased significantly over the last decade [8]. This notion is further supported by the proportion in the sample of FB and WB in the present study (35% vs 65%, respectively). Nonetheless, without context it is difficult to draw firm conclusions regarding ‘WHY’ such demands have increased. The findings of the present study demonstrated that the increased physical demands of modern WDP (e.g., WB styles) appears to be due to them actively engaging in attacking and transition phases whilst performing high-intensity ‘Support Play’ and ‘Over/Underlap’ activities when in possession, and ‘Recovery Run’ actions when dispossessed (i.e., out of possession), which is consistent with previous findings [20, 29]. This particular trend of WDP in modern European football may exist as a function of tactical evolution [20], and depends upon a team’s philosophy/tactics (e.g., how the team use WDP during a match). Therefore, applied staff should consider the playing style of their WDP within the team (e.g., FB or WB) when prescribing training drills that are tailored to the players given the substantial differences in the physical-tactical demands between them. Additionally, as the integrated approach is sensitive enough to detect specific playing styles of players with their unique physical-tactical attributes, this has some potential benefits for recruitment. For instance, players who have the physical-tactical attributes matched to the team’s playing style could be shortlisted for scouting. However, to be able to build team and positional-level physical-tactical profiles, recruitment teams need to be able to use the same level of detail presented in this study to recruit players that possess the physical qualities to execute the team’s desired tactical plan.

Unlike previous research that analysed physical metrics in isolation [4, 10], the present study demonstrated unique physical-tactical match profiles inherent in various tactical roles. In possession, COP covered more high-intensity distance for ‘Break into Box’ (ES: 0.6–2.8) and ‘Run in Behind/Penetrate’ (ES: 1.1–5.2) compared to other positions. This could be explained by offensive players attacking space in behind the opponent back line and/or entering the box to score a goal [34]. Additionally, WOP performed greater ‘Run with Ball’ distance at high-intensity (ES: 0.8–1.7) whilst WDP ran more ‘Over/Underlap’ distances (ES: 0.9–1.4) compared to other positions, both of which agree with previous findings [29, 35]. Furthermore, WDP and WOP performed more high-intensity distance for ‘Support Play’ (ES: 0.3–2.9) and ‘Move to Receive/Exploit Space’ (ES: 0.3–2.5) than CDP and CMP. It is noteworthy in that CMP covered less distance at high-intensity for ‘Support Play’ than WDP and WOP given the purpose of the action. ‘Support Play’ is when the ball is played forward quickly and the players behind or level with the ball tend to produce high-intensity efforts to become involved in the attacking/transition phase of play, which is vital to produce an offensive threat [36]. This disparity may be due to a variety of tactical roles within CMP (e.g., B2BM, CDM and CAM) in which the highest percentage spread in high-intensity running distance (32–33%) has been reported [7]. Furthermore, this could be explained with the data for specialised tactical roles, demonstrating that no differences were observed in the high-intensity distance covered for ‘Support Play’ between B2BM, CAM, WB, and WM, but all of them covered ˜670–890% greater distance than CDM.

Out of possession, COP performed more high-intensity ‘Close Down/ Press’ activities than other positions (ES: 1.1–4.2). This may be due to the increased adoption in modern football of the ‘pressing’ tactic. [37]. Nonetheless, when it comes to specialised tactical roles, CF^1^ covered ˜40% greater high-intensity distance for such actions compared to CF^2^ (˜270 m vs ˜190 m, respectively). This is possibly be due to the number of players up front as forwards since a single centre forward (e.g., forwards in a 4-5-1 formation) tends to cover greater high-intensity distance when out of possession [5] compared to two players up front (e.g., forwards in a 4-4-2 formation). In contrast, CDP covered less distance at high-intensity for ‘Close Down/ Press’ but greater for ‘Covering’ compared to other positions. This might be because CDP have limited space to achieve high-intensity running (> 19.8 km · h^-1^) to close the opponent down when defending but have more space behind them to cover space or a player whilst being goal side, especially when they are around the half line [19]. This could be confirmed by the fact that the average high-intensity distance covered by CDP for ‘Closing Down/Press’ was lower than COP (˜15 m vs ˜20 m, respectively). CDP may accelerate more to close down the opponent since maximal accelerations are often executed at velocities below high-intensity speed thresholds [38]. This suggests that the ability to frequently perform accelerations is a key requirement for CDP to be prepared for. Collectively, physical-tactical performance data clearly explains ‘WHY’ players cover the high-intensity running distance during a match. It would be more effective to use the data of specialised tactical roles when prescribing training drills whilst replicating physical-tactical demands of play since applying generalist positions could lead to the misinterpretation of the contextualised data in selected positions.

No studies in the literature have attempted to compare match running performance analysed with the general positional analysis (e.g., CMP) to that with the specialised positional analysis (e.g., B2BM, CDM, and CAM) for determining their sensitivity. The comparison of the physical-tactical characteristics between the two different analyses has revealed that the player’s physical-tactical demands of play can be under or overestimated if using the general positional analysis. When comparing FB and WB to their general role (WDP), 34% less and 15% more high-intensity distance was covered by respective tactical roles. Furthermore, the average numbers of high-intensity activities per match for ‘Recover Run’, as well as ‘Support Play’, ‘Move to Receive/Exploit Space’, and ‘Run in Behind/Penetrate’ could be overestimated for FB whilst these could be underestimated for WB except for ‘Recovery Run’ if using the general positional analysis. This trend could be due to WB running higher up the pitch to get involved in the attacking or the transition phase of play after the team regains the possession of the ball, and then producing ‘Recovery Run’ actions to get goal side when a turnover in possession occurs [20]. WB play akin to WM given that no differences were observed between them regarding all of the in-possession categories except for ‘Over/Under-lap’ and ‘Run in Behind/Penetrate’. On the other hand, CDM covered 30% less high-intensity distance compared to their general role (CMP) whilst CAM performed 22% more albeit with no statistical difference. Specifically, the average number of high-intensity ‘Close Down/Press’ actions performed by CDM (*n* = 2) and CAM (*n* = 9) per game could be over or underestimated with the use of the general positional analysis (CMP, *n* = 5) whereas CAM could perform less high-intensity ‘Covering’ actions per match. This clearly shows their different tactical duties during a match. For example, CAM are more likely to support the press whilst attackers are aggressively closing down the opponent on the ball or receiving the ball [39]; however, CDM tend to stay back to ensure defensive coverage whilst blocking space in front of the defence [24]. Moreover, in possession greater numbers of high-intensity ‘Support Play’, ‘Move to Receive/Exploit Space’, ‘Run in Behind/Penetrate’, and ‘Break into Box’ actions were executed for CAM compared to their general position (CMP); however, an opposite trend was seen for CDM. Thus, again coaches and practitioners should consider the specific tactical roles of players within the team when conditioning their players during training sessions. However, such detailed positional analysis is labour-intensive as it requires the observation of each player per match to be considered in light of numerous contextual factors that can influence match performance [4, 5]. Hence, the development and adoption of machine learning approaches will be key in automatically classifying these specialised tactical roles [24].

### Limitations

The samples of certain positions (e.g., CAM and WF) were relatively small compared to other positions, which could have affected the trends presented in this study. However, this could be due to the stringent game selection criteria for balancing and controlling data and/or such positions being more likely replaced with substitutions [40]. Moreover, as the match-to-match variability for high intensity distance (CV: 21–22%) and physical-tactical performance (CV: 62–67%) were high, the reader should always be aware that the present findings are hyper niche in relation to the EPL and the specific cultural and stylistic elements of that league, and thus may not necessarily apply to other elite leagues.

## CONCLUSIONS

Using generalist positions is less sensitive to estimate player’s actual isolated physical and contextualised demands and this may under and overestimate overall physical performance metrics of selected positions. The contextualised data trends presented could have huge practical implications for the design of positional play and position specific training sessions as well as recruitment. Finally, readers should be aware of the high degree of match-to-match variability exhibited in physical-tactical actions and understand that metric stability will be difficult to establish.

## Conflict of interest

The authors have no conflict of interest to declare.
